# Biomechanical risk factors associated with iliotibial band syndrome in runners: a systematic review

**DOI:** 10.1186/s12891-015-0808-7

**Published:** 2015-11-16

**Authors:** Jodi Aderem, Quinette A. Louw

**Affiliations:** Faculty of Medicine and Health Sciences, Physiotherapy Division, University of Stellenbosch, PO Box 19063, Francie van Zijl Drive, Tygerberg, 7505 South Africa

**Keywords:** Iliotibial band syndrome, Running, Biomechanics

## Abstract

**Background:**

Iliotibial band syndrome is the second most common running injury. A gradual increase in its occurrence has been noted over the past decade. This may be related to the increasing number of runners worldwide. Since the last systematic review, six additional papers have been published, providing an opportunity for this review to explore the previously identified proximal risk factors in more detail. The aim of this systematic review is thus to provide an up to date quantitative synthesis of the trunk, pelvis and lower limb biomechanical risk factors associated with Iliotibial band syndrome in runners and to provide an algorithm for future research and clinical guidance.

**Methods:**

An electronic search was conducted of literature published up until April 2015. The critical appraisal tool for quantitative studies was used to evaluate methodological quality of eligible studies. Forest plots displayed biomechanical findings, mean differences and confidence intervals. Level of evidence and clinical impact were evaluated for each risk factor. A meta-analysis was conducted where possible.

**Result:**

Thirteen studies were included (prospective (*n* = 1), cross-sectional (*n* = 12)). Overall the methodological score of the studies was moderate. Female shod runners who went onto developing Iliotibial band syndrome presented with increased peak hip adduction and increased peak knee internal rotation during stance. Female shod runners with Iliotibial band syndrome presented with increased: peak knee internal rotation and peak trunk ipsilateral during stance.

**Conclusion:**

Findings indicate new quantitative evidence about the biomechanical risk factors associated with Iliotibial band syndrome in runners. Despite these findings, there are a number of limitations to this review including: the limited number of studies, small effect sizes and methodological shortcomings. This review has considered these shortcomings and has summarised the best available evidence to guide clinical decisions and plan future research on Iliotibial band syndrome aetiology and risk.

## Background

Iliotibial band syndrome (ITBS) is the second most common running injury [1]. It is the main cause of lateral knee pain in runners and accounts for approximately one tenth of all running injuries [1]. An increase in ITBS was noted over the past decade and may be related to the increasing number of runners worldwide [2].

The causal pathway of ITBS is thought to be multifactorial and the underlying pathology is poorly understood [3]. A historical perspective is that ITBS is caused by excessive friction of the distal Iliotibial band (ITB) as it moves over the lateral femoral epicondyle during repetitive knee flexion and extension [4]. A more recent theory of the cause is impingement of the ITB against the lateral femoral epicondyle at approximately 20-30° of knee flexion [5, 6]. Anatomical factors such as leg length differences and increased prominence of the lateral epicondyles have also been noted as possible non-modifiable factors associated with ITBS [1, 5, 7–9]. Modifiable factors such as reduced flexibility and muscle weakness, particularly of the hip abductor muscles may also be associated with ITBS [10–14]. Unfortunately, the evidence that any of these factors are associated with the development of ITBS remains limited and inconsistent.

Biomechanical alterations may be related to ITBS in runners. The findings of two systematic reviews [15, 16] suggest biomechanical differences in runners with ITBS compared to healthy runners. van der Worp and Maarten [15] conducted a broad review of ITBS aetiology, diagnosis and treatment using a narrative method of reporting. Louw and Dreary’s [16] aim was to ascertain if there are lower limb biomechanical differences in runners with and without ITBS and used a qualitative method of data synthesis. Louw and Dreary [16] proposed that proximal segments i.e. sagittal and frontal plane motion of the hip joint, could be linked to ITBS. However, since the review by Louw and Dreary [16] six new papers which report on biomechanical factors related to ITBS were published. These additional papers provide the opportunity to explore proximal factors, as suggested by Louw and Dreary [16], in more detail. In addition, these six paper may allow for quantitative analysis on which recommendations for research and practice can be based.

The aim of this systematic review is thus to provide an up to date quantitative synthesis of trunk, pelvis and lower limb biomechanical risk factors associated with ITBS in runners, derived from prospective and cross-sectional designs. In addition, we aim to provide a succinct, user friendly summary in the format of an algorithm to assist with the design of future research and provide a guide to clinicians which is based on the currently available best evidence.

## Methods

Data from published cross-sectional and cohort studies written in English, reporting on the 3D biomechanical risk factors associated with ITBS in runners were considered for inclusion. Studies were included if they were conducted to determine whether lower limb biomechanical differences exist between runners with ITBS or those who went on to developing ITBS compared to healthy runners irrespective of gender. Studies were excluded if they were conducted on cadavers or animals.

The following medical electronic databases were searched from inception to May 2014: PubMed, Science Direct, Scopus and SPORTDiscus. A broad strategy search approach was used, using the following search terms: ((Iliotibial band syndrome OR Iliotibial band friction syndrome OR Iliotibial band strain) AND (running OR run)). The search terms were selected to maximize potential hits. In order to increase the search, Pearling (searching the reference lists of eligible and published systematic reviews) was conducted. Full text articles were retrieved for studies which were deemed potentially eligible, based on the eligibility criteria. Upon revision of the systematic review an additional search on PubMed was conducted in April 2015 using the same search criteria used in May 2014.

The reviewer (JA) and second reviewer (QL) independently screened the titles and abstracts of all initial hits and all potential full text papers according to the eligibility criteria described above. The findings of both reviewers were discussed to ensure that all possible articles were screened and identified for inclusion.

The Critical Appraisal Form for Quantitative Studies was used to appraise the methodological quality of the selected papers [17]. This tool was chosen as it gives good representation of the methodology used in quantitative research. The reviewers referred to the user guidelines to assist in interpretation of the critical appraisal tool (CAT). The second reviewer reviewed the reviewer’s results and discrepancies in findings were discussed. The CAT comprised of 16 dichotomous questions. All questions which were answered *‘yes’* added to the total score except for questions 3 and 4 where *‘no’* was positive and added to the total score. The best score for methodological quality was 16. Following the methodological appraisal, included studies were classified according to their methodological quality. Since there are no gold standards, a CAT score above 75 % was considered good methodological quality, a score between 50–75 % was considered moderate quality and a score lower than 50 % was deemed to be of poor methodological quality.

To assess consistency of diagnosis, a seven item scale diagnosis checklist was compiled by the researcher. This was based on previously used inclusion and exclusion criteria for ITBS participants [18]. Each paper was given a total score out of seven. A higher score indicated relatively better application of the inclusion and exclusion criteria.

Two customised excel spreadsheets, based on Cochrane forms were used for data extraction. These spreadsheets extracted information regarding the sample demographics as well as the study aims, gait analysis tool used, running conditions, running speed and phase of the gait cycle analysed.

The FORM framework was followed to grade available evidence and provide recommendations for clinicians to identify risk factors of ITBS [19]. The FORM framework was developed, trialed and refined between 2004–2009 to provide an expanded and revised version of the Australian NHMRC (National Health and Medical Research Council) standards to adapt to the rapid growth and diversification of clinical practice [19]. For the purpose of this study two out of the five components of the FORM framework were used. The two elements utilized included the level of evidence and the clinical impact. These elements are aligned with the aims of this systematic review.

The level of evidence refers to the quality of evidence available for each biomechanical risk factor [19]. The evidence level for each biomechanical risk factor was graded according to the NHMRC hierarchy for aetiology which can be seen in Table 1.

**Table 1:** NHMRC grading of evidence levels for aetiology

Evidence level	Study design
I	Systematic review of prospective cohort studies
II	One prospective cohort study
III	One retrospective cohort study
IV	A case control study
V	A cross-sectional study or case series

Clinical impact (effect size) is a subjective measure of the likely benefit that applying a particular finding would have on a specific population [19]. Effect size was calculated for biomechanical outcomes for which there was a significant difference found between runners with ITBS and healthy runners. The mean difference in angles between runners with ITBS and healthy runners was used to calculate effect size. A difference of 2° or more was considered clinically meaningful as a difference of less than 2° may simply be due to measurement error.

Data was described narratively using tables or narrative summaries where appropriate. A random effects model in Revman version 5.2 was used to calculate mean differences and 95 % confidence intervals (CI) provided that means and standard deviations (SD) were reported. Forest plots illustrating the mean difference and 95 % CI were generated for graphic illustration. A meta-analysis was conducted for risk factors which were reported in at least two studies, provided that homogeneity in the outcomes and samples were present with regards to gender and footwear.

## Results

**Fig. 1:**
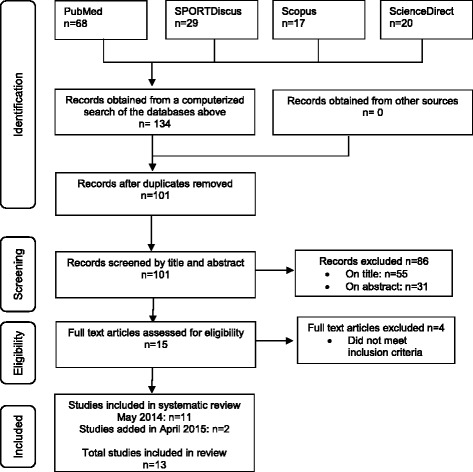
PRISMA flow diagram of literature search

The initial search in May 2014 based on the search words described above yielded a total of 134 hits. Following the application of the inclusion and exclusion criteria to the titles and the removal of duplicates, 86 studies were excluded reducing the total number of potential studies for inclusion to 46. 31 studies were excluded after abstracts were read. The primary reason for excluding these studies was because they were conducted on participants who took part in sports other than running (cycling) and because they were not conducted on or compared to participants who currently had ITBS, had previously had ITBS or went on to developing ITBS during the study. After reading the full texts the number of studies to be included in this systematic review was reduced to 11. Following an updated search in April 2015, 2 additional papers were considered eligible, resulting in 13 papers to be included in the review. Results of the search strategy can be seen in Fig. 1.

The number of participants in each study varied from 16–126. One study compared the kinetic and kinematic findings of males to females [20]. All participants were runners who ran on a weekly basis. A sample description of the thirteen eligible studies can be seen in Table 2.

**Table 2:** Sample description

	Sample size N			Gender M/F		Mean Age yrs(SD)		Mass kg(SD)		Height m(SD)		Running mileage km(w/mo)	
	TOT	ITB	CON	ITB	CON	ITB	CON	ITB	CON	ITB	CON	ITB	CON
Orchard et al. [7]	9	9	N/A	4M 5F	N/A	27.0 (9.5)	N/A	DNR	N/A	DNR	N/A	DNR	N/A
Meisser et al. [8]	126	56	70	33M 17F	53M 17F	33.9 (1.2)	35.0 (1.2)	66.4 (1.9)	70.2 (1.3)	1.7 (0.13)	1.74 (0.10)	50.3 w	42.5 w
Noehren et al. [11]	36	18	18	18F	18F	26.8	28.5	DNR	DNR	DNR	DNR	96.2 mo	99.3 mo
Ferber et al. [12]	70	35	35	35F	35F	35.47 (10.35)	31.23 (11.05)	58.62 (3.97)	61.30 (6.97)	1.65 (0.06)	1.67 (0.07)	123.82 mo	119.27 mo
Phinyomark et al. [20]	96	48	48	29F 19M	29F 19M	34.0(8)F 39.0(11)M	DNR	61.0(9)F 79.0(10)M	DNR	1.69(0.06)F 1.79(0.07)M	DNR	DNR	DNR
Foch et al. [21]	27	9 9P	9	9F 9FP	9F	26.2(7.9) 24.7(5.2)P	25.3(7.0)	53.3(3.7) 61.7(9.9)P	59.6(5.2)	1.64(0.04) 1.68(0.03)P	1.71(0.05)	34.8 w 35.2 w P	45.2 W
Foch and Milner [22]	40	20	20	20F	20F	26.0 (5.6)	23.7 (5.5)	58.8 (7.4)	58.9 (5.7)	1.67 (0.04)	1.68 (0.06)	41.8 w	38.6 W
Foch and Milner [23]	34	17	17	17F	17F	26.6 (6.6)	25.4 (6.2)	57.9 (3.9)	58.0 (4.6)	1.67 (0.05)	1.67 (0.06)	44.9 w	44.7 W
Grau et al. [24]	36	18	18	13M 5F	13M 5F	36.0 (7.0)	37.0 (9.0)	71.0 (12.0)	70.0 (10.0)	1.77 (0.08)	1.77 (0.09)	DNR	DNR
Hein et al. [25]	36	18	18	18F	18F	36.0 (7.0)	37.0 (9.0)	71.0 (12.0)	70.0 (10.0)	1.77 (0.08)	1.77 (0.09)	DNR	DNR
Miller et al. [26]^a^	16	8	8	DNR	DNR	27.5 (9.0)	26.4 (7.7)	68.7 (15.9)	71.3 (14.4)	1.7 (0.06)	1.72 (0.08)	DNR	DNR
Miller et al. [27]^a^	16	8	8	DNR	DNR	27.5 (9.0)	26.4 (7.7)	68.7 (15.9)	71.3 (14.4)	1.7 (0.06)	1.72 (0.08)	23.7 w	11.8 w
Noehren et al. [28]	34	17	17	17M	17M	33.5 (6.6)	28.1 (5.7)	76.7 (5.7)	69.9 (8.7)	1.79 (0.06)	1.80 (0.07)	31.4 w	.8 w

*Abbreviations*: *n* number of participants, *M* male, *F* female, *yrs* number of years, *SD* standard deviation, *kg* kilograms, *m* meters, *km* kilometres, *w* weekly, *mo* monthly, *TOT* total number of participants, *ITB* group of participants with ITBS, *CON* group of healthy participants, *N/A* not applicable, *DNR* did not report, *P* previous ITB

*study conducted on runners who ran to fatigue

A common aim among all studies was to determine whether there is a difference in the lower limb biomechanics of runners with ITBS or who went on to developing ITBS, compared to a control group of healthy runners. One study compared the biomechanics of female runners with ITBS to those who previously had ITBS and also to a control group [21]. In addition three of these studies also evaluated the trunk and pelvis [21–23]. Two studies included participants who ran barefoot (unshod) [24, 25], seven studies included participants who ran in a neutral running shoe (shod) [11, 12, 20–23, 28] and four studies included runners who ran in their own running shoes (shod) [7, 8, 26, 27]. Three studies evaluated the full stride cycle [20, 26, 27], the remainder evaluated the stance phase of running. A description of the study information including study aims as well as procedures can be seen in Table 3. Table 4 specifies which leg of the control group was used as a comparable to the affected leg of the ITBS group.

**Table 3:** Description of study information

	Study Aim	Gait analysis tool	Running condition	Speed	Phase of running cycle
Orchard et al. [7]	To establish a model of the pathogenesis of ITBS in distance runners	Vicon 3D Motion analysis, force plate was used	2 x 2 minute runs on a treadmill, second run was performed with a heel raise Runners own running shoes	Constant pace	Stance phase
Meisser et al. [8]	To determine whether there is a relationship between selected variables and runners affected by ITBS	High speed video camera, force plate was used	22.75m runway Runners own running shoes	Self-selected	Stance phase
Noehren et al. [11]	To compare the pre-existing frontal and transverse plane lower extremity kinetics and kinematics between a group of female runners who develop ITBS compared to healthy controls	6-camera Vicon 3D Motion analysis, force plate was used	25m runway Standard neutral running shoes	3.7m/s^−1^	Stance phase
Ferber et al. [12]	To examine differences in running biomechanics between runners who previously sustained ITBS and runners with no knee-related running injuries	6-camera Vicon 3D motion analysis, force plate was used	25m runway Neutral cushioning running shoes	3.65m/s^−1^	Stance phase
Phinyomark et al. [20]	To examine differences in running gait kinematics between male and female runners with ITBS and to assess differences in gait kinematics between healthy gender and age-matched runners compared to runners with ITBS	8-camera Vicon 3D motion analysis, no force plate was used	Treadmill Neutral running shoes (Nike Pegasus)	Self-selected speed between 2.23-3.35m/s^−1^	Full stride cycle
Foch et al. [21]	To determine if biomechanics during running, hip strength and ITB flexibility differ among female runners with ITBS, previous ITBS and controls	9-camera Vicon 3D motion analysis, force plate was used	17m runway Neutral running shoes (Bite Footwear)	3.3m/s^−1^	Stance phase
Foch and Milner [22]	To determine whether women with previous ITBS exhibited differences in kinetics and kinematics during running compared to controls using a PCA approach	9-camera Vicon 3D motion analysis, force plate was used	17m runway Neutral running shoes (Bite Footwear)	3.5m/s^−1^	Stance phase
Foch and Milner [23]	To determine if biomechanics during running and frontal plane core endurance differ between female runners with previous ITBS and controls	9-camera Vicon 3D motion analysis, force plate was used	17m runway Neutral running shoes (Bite Footwear)	3.5m/s^−1^	Stance phase
Grau et al. [24]	Investigate differences between healthy runners and runners with ITBS with regards to kinematic characteristics in order to suggest treatment strategies for ITBS	6-camera Vicon 3D motion analysis, force plate was used	13m EVA foam runway Barefoot	3.3m/s^−1^	Stance phase
Hein et al. [25]	To determine whether or not CRP variability is an effective and beneficial method for providing information about possible differences or similarities between injured and non-injured runners	6-camera Vicon 3D motion analysis, did not state whether a force plate was used	13m EVA foam runway Barefoot	3.3m/s^−1^	Stance phase
Miller et al. [26]^a^	To investigate the role of lower extremity coordination variability in runners with retrospective cases of ITBS during an exhaustive run	8-camera Vicon 3D motion analysis, no force plate used	Quinton treadmill at a level grade Runners own running shoes	Speed that would exhaust the runner within 20 minutes	Full stride cycle
Miller et al. [27]^a^	To expand the base of knowledge of ITBS biomechanics when comparing runners with ITBS to healthy runners during a run to voluntary exhaustion	8-camera Vicon 3D motion analysis no force plate used	Quinton treadmill at a level grade Runners own running shoes	Speed that would exhaust the runner within 20 minutes	Full stride cycle
Noehren et al. [28]	To assess the difference in abduction and external rotation strength, ITB length as well as frontal and transverse plane kinematics at the hip and knee in men with and without ITBS	15-camera Vicon 3D motion analysis, no force plate was used	Treadmill Neutral running shoes (New Balance WR662)	3.3m/s^−1^	Stance phase

*Abbreviations*: *m* meters, *ITBS* Iliotibial band syndrome, *3D* three dimensional, *m/s* ^*−1*^ meters per second, *PCA* Principal components analysis; ITB, Iliotibial band

^a^study conducted on runners who ran to fatigue

**Table 4:** Comparison of legs used when comparing case to control

Case (ITBS)		Control (healthy)	Source
ITBS side	vs	Right leg	Noehren et al., [11]; Ferber et al., [12] Foch et al., [21]
ITBS side	vs	Same leg	Grau et al., [24]; Hein et al [25]; Noehren et al., [28]
ITBS side	vs	Random leg	Meisser et al., [8]
ITBS side	vs	Non injured leg	Orchard et al., [7]
ITBS side	vs	Did not state	Phinyomark et al., [20]; Foch and Milner [22]; Foch and Milner [23]; Miller at al., [26]^a^; Miller et al., [28]^a^

*Abbreviations*: *ITBS* iliotibial band syndrome, *vs* versus

^a^study conducted on runners who ran to fatigue

The methodological quality appraisal scores of the thirteen eligible studies can be seen in Table 5. The mean methodological score was 62.98 %. Based on the reviewer’s classification of methodological quality, none of the thirteen studies was deemed good quality. All of the studies were considered to be of moderate quality scoring between 56.25 % – 68.75 %.

**Table 5:** Methodological quality appraisal

		Orchard et al. [7]	Meisser et al. [8]	Noehren et al. [11]	Ferber et al. [12]	Phinyomark et al. [20]	Foch et al. [21]	Foch and Milner [22]	Foch and Milner [23]	Grau et al^.^. [24]	Hein et al. [25]	Miller at al. [26]^a^	Miller et al. [27]^a^	Noehren et al. [28]
1	The purpose of the study was clearly stated	+	+	+	+	+	+	+	+	+	+	+	+	+
2	The study design was appropriate	+	+	+	+	+	+	+	+	+	+	+	+	+
3	The study detected sample biases (No adds to the total score)	+	+	+	+	+	+	+	+	+	+	+	+	+
4	Measurement biases were detected in the study (No adds to the total score)	+	+	+	+	+	+	+	+	+	+	+	+	+
5	The sample size was stated	+	+	+	+	+	+	+	+	+	+	+	+	+
6	The sample was described in detail	+	+	+	+	+	+	+	+	+	+	+	+	+
7	The sample size was justified	-	-	+	+	-	+	-	+	-	-	-	-	+
8	The outcomes were clearly stated and relevant to the study	+	+	+	+	+	+	+	+	+	+	+	+	+
9	The method of measurement was described sufficiently	+	+	+	+	+	-	+	+	+	+	+	+	+
10	The measures used were reliable	-	-	-	-	-	-	-	-	-	-	-	-	-
11	The measures used were valid	-	-	-	-	-	-	-	-	-	-	-	-	-
12	The results were reported in terms of statistical significance	+	+	+	+	+	+	+	+	+	+	+	+	+
13	The analysis methods used were appropriate	+	+	-	+	+	+	+	+	+	+	+	+	+
14	Clinical importance was reported	+	+	+	+	+	+	-	-	+	-	+	+	+
15	Missing data was reported where appropriate	-	-	+	-	-	-	-	-	+	-	-	-	-
16	Conclusions were relevant and appropriate given the methods and results of the study	+	+	-	+	+	+	+	+	+	+	+	+	+
	Study Results													
	Total CAT score /16	10	10	10	11	10	10	9	10	11	9	10	10	11
	Total CAT %	62.50	62.50	62.50	68.75	62.50	65.20	56.25	62.50	68.75	56.25	62.50	62.50	68.75

*Abbreviations*: *CAT* Critical appraisal tool

^a^ study conducted on runners who ran to fatigue

Table 6 outlines the diagnostic criteria used by the eligible studies to determine which participants were eligible to take part. Eligible studies used these criteria to determine participant inclusion.

**Table 6:** Diagnostic criteria results for ITBS

	Key inclusion and exclusion criteria	Orchard et al. [7]	Meisser et al. [8]	Noehren et al. [11]	Ferber et al. [12]	Phinyonmark et al. [20]	Foch et al. [21]	Foch and Milner [22]	Foch and Milner [23]	Grau et al. [24]	Hein et al. [25]	Miller et al. [26]^a^	Miller et al. [27]^a^	Noehren et al. [28]
1	Clear definition of location of pain was reported	✓	✓	X	✓	✓	✓	X	X	✓	✓	X	X	✓
2	Reports a typical history of ITBS with symptoms consistent to the condition	✓	X	X	✓	✓	✓	X	X	✓	✓	X	✓	✓
4	Diagnosis was confirmed by a medical practitioner/physiotherapist/ trainer	✓	✓	✓	✓	✓	✓	✓	✓	✓	✓	✓	✓	✓
4	A positive clinical test (Obers/Nobles)/ palpation	✓	✓	X	X	✓	X	X	X	✓	✓	X	✓	✓
5	No previous knee surgery	✓	X	✓	✓	✓	X	✓	✓	✓	✓	✓	X	✓
6	No internal derangement or other sources of lateral knee pain present	✓	✓	✓	✓	✓	X	✓	✓	✓	✓	✓	X	✓
7	No previous spine or lower limb injury	✓	✓	✓	✓	✓	X	X	✓	✓	✓	✓	X	✓
Criteria’s Met		7	5	4	6	7	3	3	4	7	7	4	3	7

*Abbreviations*: *ITBS* Iliotibial band syndrome

^a^ study conducted on runners who ran to fatigue

Ten of the thirteen studies evaluated the stance phase of running [7, 8, 11, 12, 21–25, 28]. Eight reported on means and standard deviations [7, 8, 11, 12, 21, 23, 24, 28], one used continuous relative phase (CRP) [25] to describe the relationship of one joint to another and one used principal components analysis (PCA) [22].

**Fig. 2:**
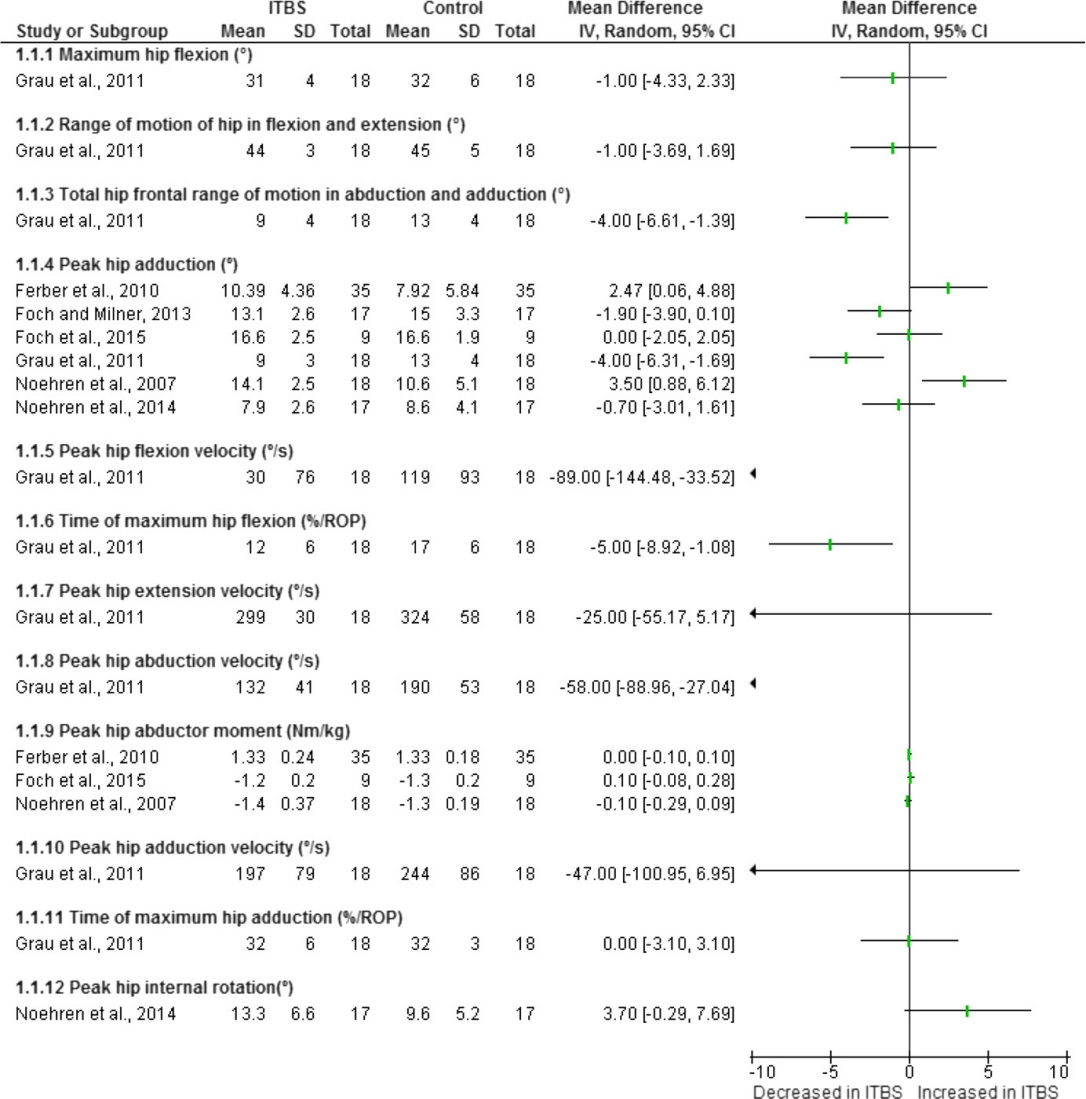
Hip risk factors during the stance phase of running in runners with ITBS

Figure 2 illustrates the hip risk factors identified during the stance phase of running in runners with ITBS. A total of twelve risk factors were studied. One study found that female shod runners who later developed ITBS had significantly increased peak hip adduction range of motion [11]. Studies which reported data on combined gender, found significantly decreased: total hip frontal range of motion in abduction and adduction [24], peak hip adduction [24], peak hip flexion velocity [24], time of maximum hip flexion [24] as well as decreased peak hip abduction velocity [24] in unshod runners with ITBS.

**Fig. 3:**
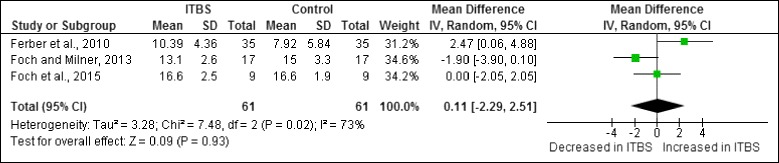
Meta-analysis of peak hip adduction (°) in female shod runners during the stance phase of running

**Fig. 4:**
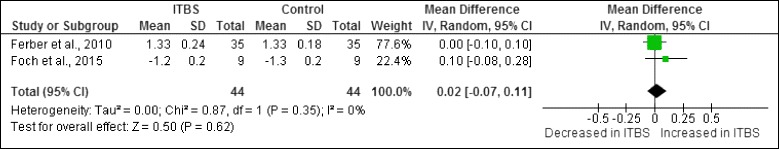
Meta-analysis of peak hip abductor moment (Nm/kg) in female shod runners during the stance phase of running

A meta-analysis was possible for two hip risk factors obtained from cross-sectional studies in females. The meta-analysis indicated that both peak hip adduction (Fig. 3) as well as peak hip abductor moment (Fig. 4) were not significantly different in female shod runners with ITBS compared to healthy runners.

**Fig. 5:**
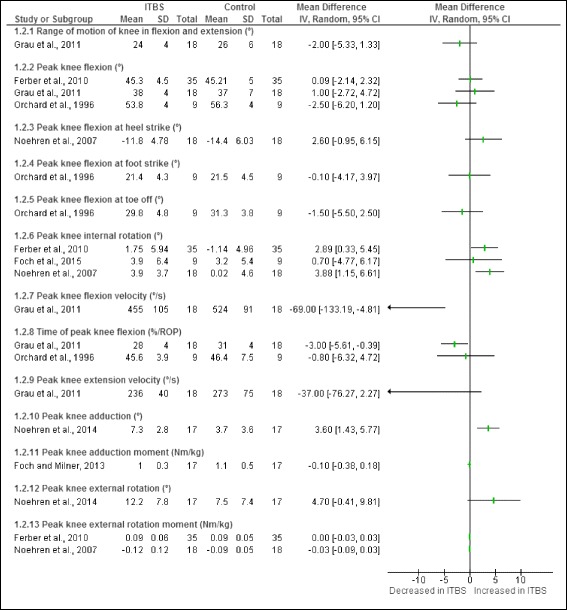
Knee risk factors during the stance phase of running in runners with ITBS

Figure *5* illustrates the knee risk factors identified during the stance phase of running in runners with ITBS. A total of thirteen risk factors were studied. One study found that female shod runners who later developed ITBS had significantly increased peak knee internal rotation range of motion [11]. One study found that female shod runners with ITBS had significantly increased peak knee internal rotation [12]. One study found that male shod runners with ITBS had increased peak knee adduction [28]. A study reporting on combined gender found unshod runners with ITBS had significantly decreased peak knee flexion velocity [24] and time of peak knee flexion [24].

**Fig. 6:**
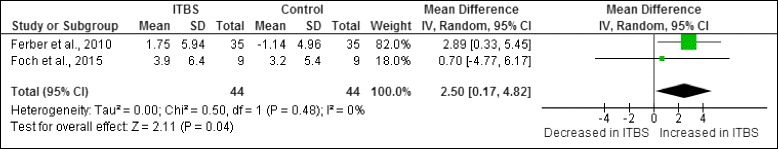
Meta-analysis of peak knee internal rotation (°) in female shod runners with ITBS during the stance phase of running

A meta-analysis was only possible for one of the knee risk factors obtained from the cross-sectional studies. This meta-analysis indicated that peak knee internal rotation was significantly increased in female shod runners with ITBS compared to healthy runners (Fig. 6).

**Fig. 7:**
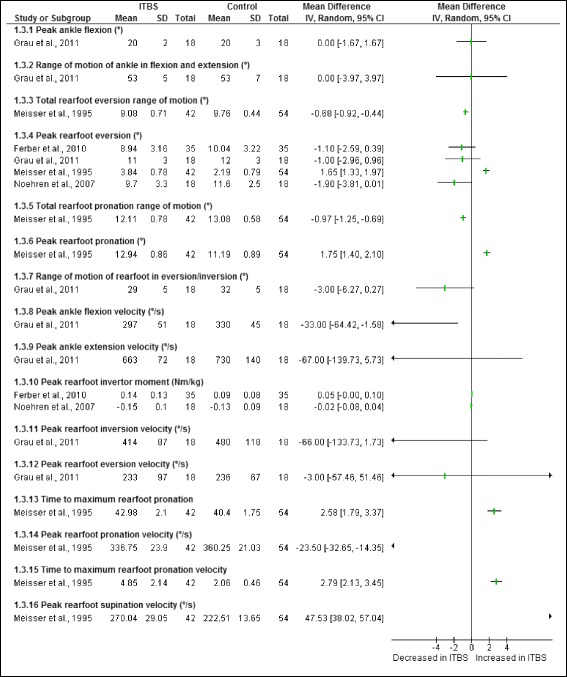
Ankle and foot risk factors during the stance phase of running in runners with ITBS

Figure 7 illustrates the ankle and foot risk factors during the stance phase of running in runners with ITBS, a total of sixteen risk factors were studied. A combined group of male and female shod runners with ITBS were found to have significantly decreased: total rearfoot eversion range of motion [8], total rearfoot pronation range of motion [8], peak ankle flexion velocity [24] and peak rearfoot pronation velocity [8]. A combined group of male and female shod runners with ITBS were also found to have significantly increased: peak rearfoot eversion [8], peak rearfoot pronation [8], peak rearfoot supination velocity [8] as well as increased time to maximum rearfoot pronation [8] and increased time to maximum rearfoot pronation velocity [8].

A meta-analysis was not possible for any of the ankle risk factors obtained from cross-sectional studies as the sample populations were not homogenous.

**Fig. 8:**
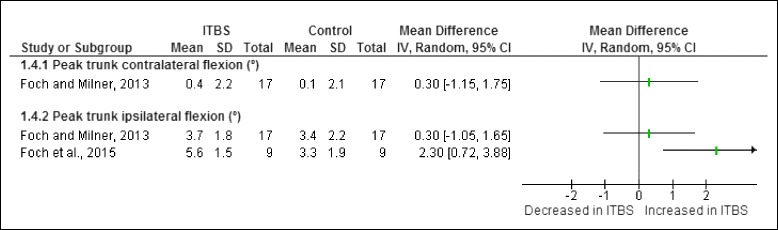
Trunk risk factors during the stance phase of running in runners with ITBS

Figure 8 illustrates the two trunk risk factors studied during the stance phase of running in runners with ITBS. One study found that female shod runners with ITBS had significantly increased peak trunk ipsilateral flexion compared to healthy runners [21].

**Fig. 9:**
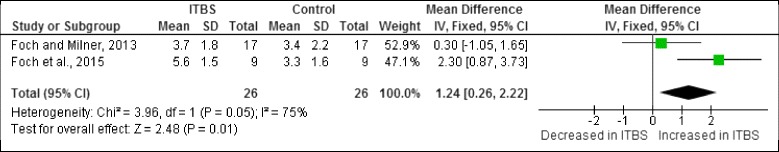
Meta-analysis of peak trunk ipsilateral flexion (°) in female shod runners with ITBS during the stance phase of running

A meta-analysis was only possible for one trunk risk factor obtained from cross-sectional studies. The meta-analysis indicated that peak trunk ipsilateral flexion is significantly increased in female shod runners with ITBS compared to healthy runners (Fig. 9).

**Fig. 10:**
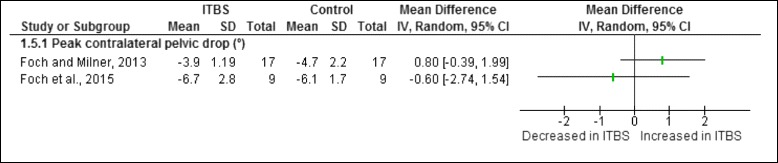
Pelvic risk factor during the stance phase of running in runners with ITBS

Figure 10 illustrates the one pelvic risk factor analysed during the stance phase of running. This risk factor was not found to be significant in female shod runners with ITBS.

**Fig. 11:**
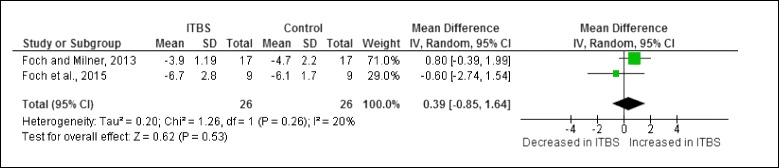
Meta-analysis of peak contralateral pelvic drop (°) in female shod runners with ITBS during the stance phase of running

A meta-analysis was possible for the pelvic risk factor obtained from cross-sectional studies. The meta-analysis indicated that peak contralateral pelvic drop is not significant in female shod runners with ITBS compared to healthy runners (Fig. 11).

Three studies were conducted on the full stride cycle [20, 26, 27].

Effects on fatigue: Two studies compared the biomechanics of shod runners with ITBS to healthy runners’ pre and post fatigue [26, 27]. Miller et al. [27] found significant differences with regards to maximum knee flexion, maximum foot adduction and peak ankle extension velocity at the beginning of the run as well as maximum knee flexion, maximum knee internal rotation velocity, maximum foot inversion and maximum ankle extension velocity at the end of the run. Miller et al. [26] used CRP to display their results and suggested that shod runners prone to ITBS may use abnormal segmental coordination patterns particularly with couplings involving thigh adduction/abduction and tibial internal/external rotation.

Gender differences: One study [20] used PCA to evaluate the differences in the kinematics of male and female shod runners with ITBS. Significant differences for hip external rotation were found for male and female runners with and without ITBS at 52-54 % of the running cycle (swing phase) as well as at 56-58 % of the running cycle (swing phase) in female runners with and without ITBS. Ankle internal rotation at 70-72 % of the running cycle (swing phase) was found to be significant when comparing the kinematics of male runners with ITBS to those who were healthy. Phinyomark et al. [20] suggests that gender should be taken into account when investigating the biomechanical cause of ITBS.

**Fig. 12:**
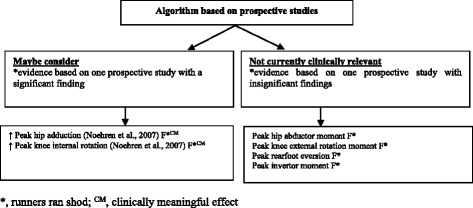
Algorithm of ITBS risk factors to screen in runners, based on evidence from prospective cohort studies

**Fig. 13:**
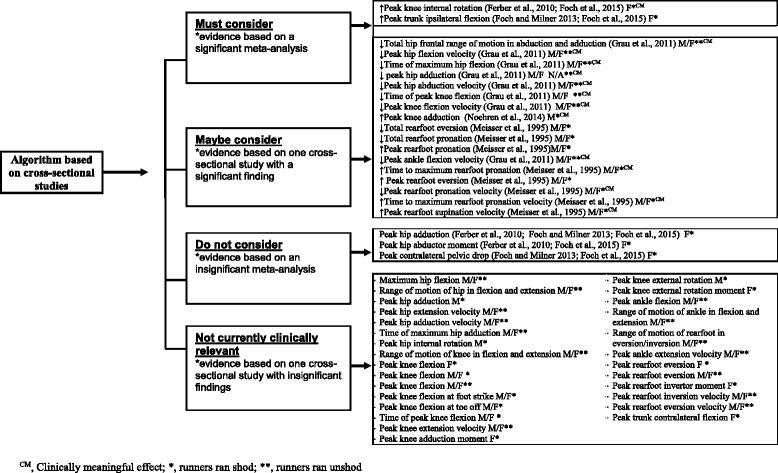
Algorithm of ITBS risk factors in runners with ITBS, based on evidence from cross-sectional studies

The FORM framework was used to evaluate the evidence of the eight studies represented in the forest plots. All studies were cross-sectional with level V evidence apart from one study of level II evidence [11]. Grading the evidence allowed for the development of an algorithm to inform future research and provide a succinct synthesis to clinicians of the current evidence base for ITBS risk factors in runners (Fig. 12 and Fig. 13). This algorithm acts as a guide for researchers/clinicians to identify the biomechanical risk factors which may be at fault in runners already presenting with ITBS or in runners who may be at risk of developing ITBS.

Findings of the single prospective study (level II evidence) on female shod runners who went onto developing ITBS [11] were classified into one of two categories, which were based on the significance of evidence (Fig. 12). Clinical impact was also stated for significant findings. Two risk factors were identified as risk factors which should ‘maybe be considered’ as these were based on only one study with a significant finding. These risk factors include peak hip adduction and peak knee internal rotation. Four risk factors were found to be insignificant and therefore ‘not currently clinically relevant’. Effect size was calculated to determine the clinical impact for the two significant risk factors identified in the category ‘maybe consider’. These two risk factors were identified as being clinically meaningful.

The findings of the seven cross-sectional studies (Level V evidence) were categorized according to one of four categories which were based on the significance of evidence (Fig. 13). To allow for comparison, findings were separated into the gender studied and whether runners ran shod or unshod. Clinical impact was also stated for significant findings. A meta-analysis was done where possible. Two risk factors were identified as factors which ‘must be considered’ as the evidence base for these risk factors was based on a significant meta-analysis of a homogenous population. These risk factors include peak knee internal rotation and peak trunk ipsilateral flexion in female shod runners. Seventeen risk factors were identified as risk factors which should ‘maybe be considered’ as these were based on only a single study with a significant finding. Three risk factors including: peak hip adduction, peak hip abductor moment and peak contralateral pelvic drop in female shod runners, were found to be risk factors which ‘do not need to be considered’ as the evidence was based on an insignificant meta-analysis. Twenty eight risk factors were found to be insignificant and therefore ‘not currently clinically relevant’. Effect size was calculated to determine the clinical impact for the two risk factors identified in the category ‘must consider’ and the seventeen significant risk factors identified in the category ‘maybe consider’. Fourteen of these risk factors were identified to be clinically meaningful.

## Discussion

The findings of our review indicate that the new evidence derived from the six additional publications since the last published review [16] have provided more insight into the biomechanical risk factors associated with ITBS, particularly about the trunk and pelvis, which was not addressed in the previous review. The finding of our quantitative analysis from cross-sectional studies showed that female shod runners with ITBS appear to have increased peak knee internal rotation and increased peak trunk ipsilateral flexion during the stance phase of running compared to healthy runners. The meta-analyses for peak hip adduction, peak hip abductor moment and peak contralateral pelvic drop between female shod runners with ITBS and healthy runners were insignificant (Fig. 13). At this stage we cannot make conclusive clinical recommendations, even for peak knee internal rotation and peak trunk ipsilateral flexion, due to the limited number of studies, small effect sizes and methodological shortcomings. The evidence for factors that may predispose runners to the development of ITBS remains limited to a single study which indicated that female shod runners who went onto developing ITBS had increased peak hip adduction and increased peak knee internal rotation during the stance phase of running. Despite these shortcomings, our review summarised the best *available* evidence to guide clinical decisions or plan future research.

Clinicians should ‘maybe consider’ screening for increased knee internal rotation and hip adduction to prevent the development of ITBS among female shod runners (Fig. 12). Due to the proximal origin of the ITB at the hip and its distal insertion onto Gerdys tubercle at the knee [29], patterns of increased hip adduction and knee internal rotation may increase the amount of strain and tension on the ITB [6]. The ITB assists in hip abduction and is stretched in adduction [30]. Increased hip adduction and knee internal rotation may be due to: weak/poor neuromuscular control of the hip abductor muscles, hip/knee joint stiffness, myofascial restrictions of surrounding musculature or altered somatosensory control. Although this proposed causal pathway is plausible, the study by Noehren et al. [11] included a small sample, with large inter-subject variation in performance (based on reported standard deviations), the researchers excluded outliers from the data analysis (influencing the validity of the study findings) and participants were not re-tested at the end of the study to ascertain whether these biomechanical differences remained present. Therefore, further research is needed for affirmation.

We included twelve cross-sectional studies [7, 8, 12, 21–28], but the evidence base for the majority of risk factors was limited to a single study. The findings from the meta-analyses showed that female shod runners with ITBS may present with increased peak knee internal rotation [12, 21] and increased peak trunk ipsilateral flexion [21, 23]. Although this presents the best evidence to date, clinicians should note that the difference between groups for peak knee internal rotation was 2.5 degrees [12, 21] and for peak trunk ipsilateral flexion was 1.24 degrees [21, 23]. This is arguable larger or the same as the likely measurement error of around two degrees of 3D motion analysis systems [31]. These small differences may thus not be clinically meaningful as it could simply reflect measurement error. Although clinicians must consider these factors in clinical practice (as it reflects the current best available evidence), clinical reasoning should still play a vital role when making clinical decisions for runners with ITBS.

We also noted that biomechanical outcomes (peak knee flexion, time of peak knee flexion and peak rearfoot eversion) may depend on whether runners wore shoes or ran barefoot during a trial capture. In addition, differences between shoes will also have an effect on the biomechanical outcomes [8, 12]. Clinicians performing gait analysis should thus consider the type of shoe and whether shoes should be worn during the assessment. This is an important recommendation for re-assessment of the same runner as it could have an effect on the results of the gait analysis test.

It was noted that many of the cross-sectional studies included in our algorithm were conducted on a combination group of males and females which made it impossible to extrapolate for which gender the findings were most applicable and made it difficult to compare findings between genders. Future studies should report data on male and females separately so that subgroup analyses can be conducted. This is required before specific clinical recommendations can be formulated.

The effect of fatigue on runners with ITBS was only evaluated by two studies. Significant differences were noted with regards to maximum knee flexion, maximum foot adduction and peak ankle extension velocity at the beginning of the run as well as maximum knee flexion, maximum knee internal rotation velocity, maximum foot inversion and maximum ankle extension velocity at the end of the run [27]. Another study showed that runners prone to ITBS may present with abnormal segmental coordination patterns particularly with couplings involving thigh adduction/abduction and tibial internal/external rotation [26]. This indicates that fatigue may have an effect on runners with ITBS, however the amount of evidence is limited. These results were not illustrated in the algorithm due to limited evidence and lack of comparability. This review acknowledges that fatigue may be considered as a risk factor of ITBS however further studies need to be conducted on CRP, PCA and fatigue to allow for further analysis and comparison with existing studies.

### Implications for Future Research

The most important finding of our review is that we identified many methodological factors which should be addressed in future research. Our review highlights key areas which should be addressed in order to advance our understanding of ITBS. Firstly, the diagnostic criteria table indicates that the only criteria to diagnose ITBS for all studies was based on whether or not a health practitioner had diagnosed the runner with having ITBS. The differences in inclusion and exclusion criteria for ITBS indicate that there may be differences in how runners were diagnosed with ITBS, which introduced heterogeneity. Although the most common diagnostic criteria was if a health practitioner had diagnosed ITBS, the interpretation of how they may have diagnosed it could have been different. Many of the studies excluded runners with knee internal derangement when diagnosing ITBS. However it was not noted which special tests of the knee were done in order to state that the runner had internal derangement of the knee. Differences in how ITBS was diagnosed indicates that international consensus to diagnose ITBS is required.

The key methodological shortcomings of the included studies were similar across the studies included in our review. All studies included convenient sampling, which limits generalizability of findings and should particularly be addressed in future cross-sectional studies. Less than 40 % of the studies justified the sample size and consequently statistical power was arguably too low to detect statistical significant differences between groups. Our concise, quantitative presentation of the data presented in this review could assist future researchers with the data required to calculate sample sizes.

None of the studies reported on the reliability and validity of the testing procedures. Although, the hardware of the widely used 3D biomechanical systems are extremely reliable, it requires some human interaction (e.g. marker placement) which introduces opportunities for measurement errors. This is very important, particularly for outcomes such as knee internal rotation, which is sensitive to marker placement errors (affecting peak knee internal rotation angle). In addition, knee rotation range is also vulnerable to soft tissue artefacts which may in fact be larger than the physiological range of knee internal rotation. Hence, the measurement error of knee rotation could be larger than the physiological range of knee rotation. Knee rotation may play a role in the development of ITBS. Future studies should thus report reliability and measurement errors to understand the attributable role of potential risk factors associated with ITBS.

This review showed that many biomechanical risk factors were analysed in the eligible studies. An astounding number of 44 risk factors were reported. It is proposed that future studies should consider published risk factors in order to compare across studies. A physiological plausible theory for selected risk factors is also lacking and this should be addressed in future studies. Increased homogeneity between studies will allow for more convincing meta-analyses which could provide guidance for clinical practice.

### Review limitations

A language bias is likely as we only considered studies published in the English language. Only two reviewers appraised the methodological quality of the papers, additional reviewers should have been used. The breakdown of the methodological appraisals was not indicated and should be included in future studies. Only one study used high speed video cameras to capture the running biomechanics which could have introduced bias. In addition, heterogeneity was introduced as not all researchers used the same diagnostic criteria for ITBS.

## Conclusion

The evidence for factors that may predispose runners to the development of ITBS remains limited to a single prospective study. This study indicated that female shod runners who went onto developing ITBS may present with increased peak hip adduction and increased peak knee internal rotation during the stance phase of running. Based on meta-analyses of cross-sectional studies, we found that female shod runners with ITBS may present with increased peak knee internal rotation and trunk lateral ipsilateral flexion during the stance phase of running. The meta-analyses of three cross-sectional studies showed no difference in peak hip adduction, peak hip abductor moment and peak contralateral pelvic drop between female shod runners with ITBS and healthy runners. However, unless the methodological rigour of ITBS research is enhanced, conclusive clinical recommendations are not possible. Future research should report reliability, validity and measurement error of methods, apply transparent data analysis approaches and include defensible sampling methods to ensure that the findings are generalizable. We also recommend international consensus on the diagnostic criteria for ITBS in future research.

## Abbreviations

CAT: Critical appraisal tool
CI: Confidence interval
CON: Control
CRP: Continuous relative phase
DNR: Did no report
F: Females
ITB: Iliotibial band
ITBS: Iliotibial band syndrome
JA: Jodi Aderem
Kg: Kilograms
Km: Kilometres
M: Males
M/F: Males and females
m: Meters
mo: Monthly
n: No
n: Total number of sample
N/A: Not applicable
NHMRC: National Health and Medical Research Council
PCA: Principle component analysis
QL: Quinette Louw
Nm: Newton’s
RCT: Randomized control trial
ROP: Roll over process
SD: Standard deviation
TOT: Total
Vs: Versus
W: Weekly
Y: Yes
Yrs: Number of years
3D: Three dimensional
